# Immunological reaction to magnesium-based implants for orthopedic applications. What do we know so far? A systematic review on *in vivo* studies

**DOI:** 10.1016/j.mtbio.2022.100315

**Published:** 2022-06-09

**Authors:** Omer Suljevic, Stefan F. Fischerauer, Annelie M. Weinberg, Nicole G. Sommer

**Affiliations:** Department of Orthopedics and Traumatology, Medical University of Graz, Graz, Austria

**Keywords:** Immunology, Mg, Implants, Inflammation, Orthopedics, Bone, Mg, Magnesium, FBGCs, Foreign Body Giant Cells, FBR, Foreign Body Reaction, SYRCLE, Systematic Review Centre for Laboratory Animal Experimentation

## Abstract

Magnesium-based implants (Mg) became an attractive candidate in orthopedic surgery due to their valuable properties, such as osteoconductivity, biodegradability, elasticity and mechanical strength. However, previous studies on biodegradable and non-biodegradable metal implants showed that these materials are not inert when placed *in vivo* as they interact with host defensive mechanisms. The aim of this study was to systematically review available *in vivo* studies with Mg-based implants that investigated immunological reactions to these implants. The following questions were raised: Do different types of Mg-based implants in terms of shape, size and alloying system cause different extent of immune response? and; Are there missing links to properly understand immunological reactions upon implantation and degradation of Mg-based implants? The database used for the literature research was PubMed (U.S. National Library of Medicine) and it was undertaken in the end of 2021. The inclusion criteria comprised (i) *in vivo* studies with bony implantation of Mg-based implants and (ii) analysis of the presence of local immune cells or systemic inflammatory parameters. We further excluded any studies involving coated Mg-implants, *in vitro* studies, and studies in which the implants had no bone contact. The systematic search process was conducted according to PRISMA guidelines. Initially, the search yielded 225 original articles. After reading each article, and based on the inclusion and exclusion criteria, 16 articles were included in the systematic review. In the available studies, Mg-based implants were not found to cause any severe inflammatory reaction, and only a mild to moderate inflammatory potential was attributed to the material. The timeline of foreign body giant cell formation showed to be different between the reviewed studies. The variety of degradation kinetics of different tested implants and discrepancies in studies regarding the time points of immunological investigations impair the conclusion of immunological reactions. This may be induced by different physical properties of an implant such as size, shape and alloying system. Further research is essential to elucidate the underlying mechanisms by which implant degradation affects the immune system. Also, better understanding will facilitate the decision of patients whether to undergo surgery with new device implantation.

## Graphical abstract

**Figure:**
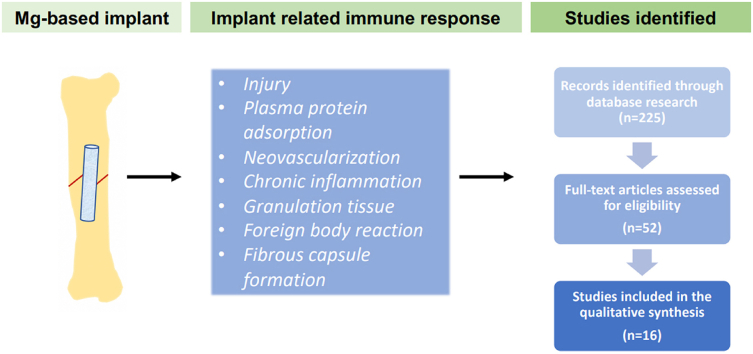

## 1 Introduction

Biodegradable implants have recently acquired attention in biomaterial research due to their favorable characteristics [1]. Magnesium (Mg)-based implants are attractive candidates in orthopedic and trauma surgery, due to their valuable properties such as osteoconductivity, biodegradability, elasticity and mechanical strength [[1], [2], [3], [4]]. After successful osteosynthesis with biodegradable Mg alloys, the implant degrades and releases Mg ions supporting new bone growth [5]. This was shown by high bone mineral apposition rates around degrading Mg-based implants and increased bone mass [6]. In addition, there are already reports of Mg-based implants being used in clinical practice, such as in fixation of hallux fractures and medial malleolus fractures [7,8]. However, biodegradable and permanent biomaterials are not inactive after implantation and they interact with host defensive mechanisms. This was demonstrated by studies that investigated biocompatibility of biodegradable and non-biodegradable implants (e.g. titanium, nickel, cobalt, poly-*l*-lactic, polyglycolic acid, pure Mg) [[9], [10], [11], [12], [13], [14], [15], [16], [17]]. Furthermore, it was reported that different metals and alloys (e.g. different additives added to Mg in order to reduce the rate of degradation) can induce different degrees of immune response without emerging from biocompatibility boundaries [[18], [19], [20], [21]]. The most common method for evaluating an implant's biocompatibility is actually the histological evaluation of the tissue adjacent to the implant, whereas blood biochemistry analysis can diagnose systemic inflammatory reactions in clinical circumstances [22].

**Fig. 1:**
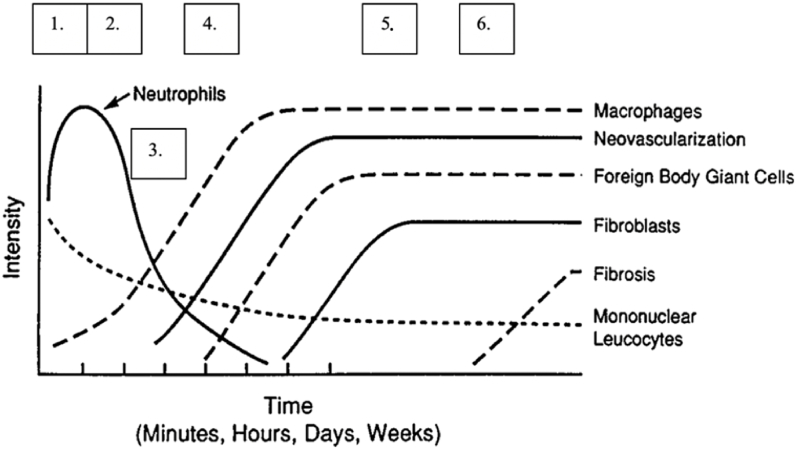
Timeline of inflammatory response to tissue implanted biomaterials [28]; 1. Injury, edema/vascular leakage, blood-material interaction and initiation of the inflammatory response, 2. Plasma proteins adsorption to material, provisional matrix formation, acute inflammation, 3. Neovascularization, 4. Chronic inflammation, 5. Granulation tissue formation, foreign body reaction, 6. Fibrous capsule formation. Adapted with permission [22]. Copyright 2001, Annual Review of Materials Research.

Biocompatibility of an implant is defined as its ability to perform with an appropriate host response in a specific application and biocompatibility assessment [22]. Shortly after implant placement and tissue injury, a cascade of events is initiated involving blood-material interaction, provisional matrix formation, acute and chronic inflammation, granulation, foreign body reaction and fibrous capsule formation (Fig. 1) [[22], [23], [24], [25], [26], [27]].

During blood-material interaction, there is a protein adsorption to the biomaterial surface with formation of a blood-based transient provisional matrix around the implant [22]. This matrix consists of cytokines, growth factors and chemo-attractants which are able to engage cells of the innate immune system [22]. The next stage is an acute inflammatory response, which is initiated by innate immunity and depends on the degree of injury [22]. This stage is driven by neutrophils (polymorphonuclear leukocytes) that secrete inflammatory cytokines resulting in the attraction of monocytes, which differentiate into macrophages [[22], [23], [24], [25], [26], [27]]. Furthermore, mast cells at the implantation site degranulate and induce histamine, interleukin-3 (IL-3) and IL-4 release to regulate the extent of foreign body reaction in a later stage [27]. The presence of monocytes, macrophages and lymphocytes with proliferation of blood vessels and connective tissue implicates the chronic inflammatory stage [22]. Macrophages are classified upon their polarization. While M1 macrophages (pro-inflammatory) are classically activated and initiate an immune response, M2 macrophages (anti-inflammatory) are alternatively activated and are associated with wound healing and tissue repair [29]. Both types promote tissue repair by secretion of cytokines and chemokines, but their exact interaction with biomaterials is not yet elucidated [30].

**Fig. 2:**
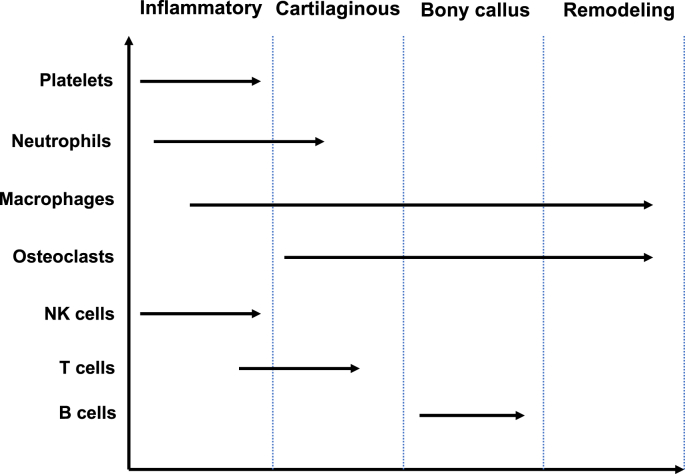
Timeline of immune cells presence during fracture healing phases. 1. Inflammatory phase, 2. Cartilaginous phase, 3. Bony callus phase, 4. Remodeling phase. Adapted with permission [41]. Copyright 2018, Current Osteoporosis Reports.

T-lymphocytes are attracted by cytokines including IL-1, TNF-α, IL-6 and IL-8 and play a major role in the polarization of macrophages [31]. They release IL-1 and IL-3 which further induce fusion of biomaterial-adherent macrophages into foreign body giant cells (FBGCs) [27,29]. However, T-lymphocytes have been associated with metal hypersensitivity (allergy caused by exposure to released metal ions often reported in permanent implants) which is probably a Type IV (delayed hypersensitivity) reaction [32]. It is believed that these released metal ions behave as haptens which bind to internal proteins and act as antigens presented to T-lymphocytes, as they are too small for inducing an immunological response on their own [[32], [33], [34]]. In contrast, B-lymphocytes showed to be activated in tissues associated with failing metal implants [35]. Certainly, it is believed that B-lymphocytes produce antibodies to the previously mentioned haptens and mediate hypersensitivity-associated reactions [32,36]. However, up to now, the detailed role of B-lymphocytes in metal implants is still undefined [[37], [38], [39], [40]]. Three to five days following biomaterial implantation, proliferation of fibroblasts with neovascularization indicates the formation of granulation tissue, which is separated from the implant by the components of the foreign body reaction (FBR) [22]. During FBR, macrophages form on the implant's surface to probably mediate oxidative damage to the implant's surface [27]. The lifetime of FBGCs on the implant surface and the correlation of FBR composition with the implant size, shape and surface is not completely understood, however, it is suggested that FBGCs are prone to stay on the implant surface for its lifetime [22]. The most prominent FBGCs are bone resorbing cells – osteoclasts [27]. However, in comparison to other FBGCs that are found in pathological conditions, osteoclasts are located at the bone surface where they cooperate with osteoblasts in the process of bone remodeling and play an important role during fracture healing phases (Fig. 2) [27].

The end stage of inflammatory reactions to implants is fibrous encapsulation. Formation of a fibrous capsule at the end disconnects the interaction of the implant with surrounding tissue [27,42]. Recent studies indicate a possible link of fibrous encapsulation with sclerotic bone rim formation around implants, however, further studies are needed for complete elucidation of this phenomenon [43,44].

The aforementioned events are considered to be a normal response of tissue to an implant placement. However, abnormalities in this process can cause different complications such as non-union, osteolysis, necrosis, fibrosis, fibrous capsule contractions, hypersensitivity or even cancer [24,45,46]. Therefore, tissue response to any implant needs to be deeply investigated [47]. To the best of our knowledge, there is no systematic review of *in vivo* studies regarding the inflammatory effect of biodegradable Mg-based implant. Hence, the aim of this study was to investigate the possible discrepancies in the rate of host response to Mg-based implants in *in vivo* studies. Also, this review will serve as a tool for understanding what is known so far about host responses to biodegradable implants, which will in the end contribute to the overall comprehension of their *in vivo* behavior.

## 2 Materials and methods

### 2.1 Objectives

The purpose of this study was to systematically review available literature regarding the immunological reaction of a living organism after implantation of biodegradable Mg-based implants. The following questions were raised and will be discussed:

• Do different types of Mg-based implants, in terms of, e.g., shape, size, and alloying system, cause a different extent of immune response?
• Are there missing links to properly understand immunological reactions upon implantation and degradation of Mg-based implants?

### 2.2 Standard criteria and type of study

This systematic review followed the PRISMA Statement suggestion on systematic review [48].

### 2.3 Eligibility criteria

#### 2.3.1 Search strategy

**Fig. 3:**
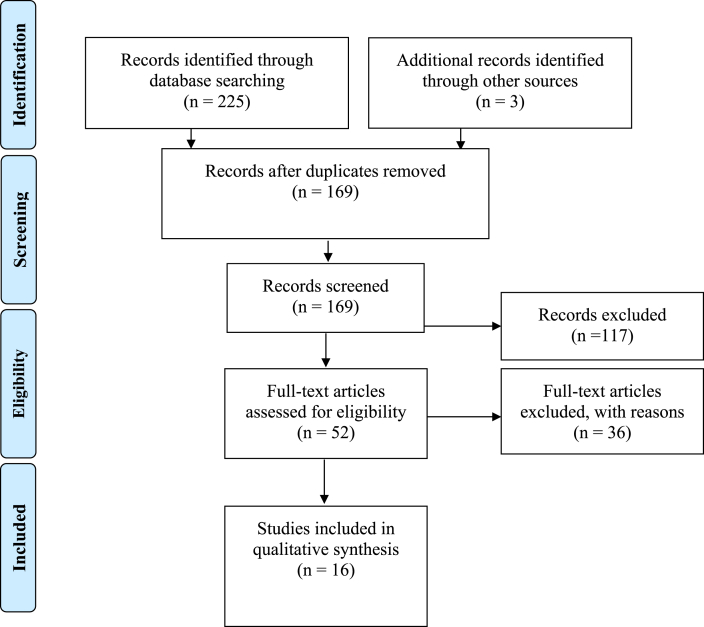
Study screening process – shows flowchart of the studies that were selected for the systematic review [48].

The database used was Medline/PubMed website (U.S National Library of Medicine, National Institutes of Health). The search was carried out for all articles published from 1.1.2010 until 31.12.2021 (Fig. 3). It was based only on the articles in the English language and the keywords searched were: magnesium implants immunology, magnesium implants macrophages, magnesium implants inflammatory, magnesium implants neutrophils, magnesium implants foreign body reaction and magnesium implants fibrous capsule. There was no contact with any of the authors.

#### 2.3.2 Inclusion criteria

All of the studies that minutely investigated the presence of immune cells as a result of immunologically driven responses after insertion of biodegradable Mg-based implants into bone were included. Implant types that were considered eligible for inclusion criteria were cylinders, screws, nails, pins and rods. There was no limitation on sample size. Regarding histological specimens, there was no exclusion based on whether it was bone, bone marrow or soft tissue surrounding the implant interface specimen.

#### 2.3.3 Exclusion criteria

We excluded all *in vitro* studies, as well as the *in vivo* studies that investigated other biodegradable alloys in which Mg was not the most abundant material; studies that implanted the material completely in soft tissue with no bone contact (due to different immunological microenvironment differences between the bone and soft tissue and difference in extent of tissue damage after soft tissue vs. orthopedic surgical procedures); studies that exposed the implants to microbes and coatings, and; studies with only the abstract available.

### 2.4 Extracted variables

From each identified article, the following data was extracted: authors, year and length of the study, tested implant type, size, material and location of insertion, type of interface tested by histology, animal species, methods used for acquiring immunological/inflammatory parameters, and type of evaluated parameters (Table 1).

### 2.5 Risk of bias assessment for animal studies

Animal *in vivo* studies that were included in the qualitative synthesis were assessed for risk of bias by using Systematic Review Centre for Laboratory Animal Experimentation (SYRCLE) tool based on Cochrane Risk of Bias tool (Table 2) [49].

## 3 Results

### 3.1 Immunological response events highlighted by evaluated *in vivo* studies in Table 1

In 2010, Castellani and colleagues implanted Mg–Y-Nd-HRE and titanium implants in femoral bones of 72 rats. They reported that differential blood count analysis did not show any systemic inflammatory reaction in the Mg–Y-Nd-HRE group. Also, there was no statistically significant difference in the percentage of lobulated neutrophil granulocytes, stab neutrophils, lymphocytes, eosinophil or basophil granulocytes in the blood count when compared with the titanium control group. However, the percentage of monocytes in the blood was significantly lower when compared to rats with Ti-alloys 24 weeks after the implantation. Also, the IL-6 enzyme-linked immunosorbent assay showed normal levels. Furthermore, histological sections showed no evidence of fibrous tissue or inflammatory reactions in both of the tested implants [50].

**Table 1:** Summary of animal *in vivo* studies which evaluated immunological parameters after Mg-based biomaterials implantation.

**Article author**	Year	Implant material/shape/size	Animal species and number	Implantation site	Histology specimen	Study length	Methods used for testing immunological reaction	Parameters evaluated
**Castellani et al.** [50]	2010	Mg–Y-Nd-HRE (Titanium group as control) Shape: cylindrical Diameter: 1.6 ​mm Length: 7 ​mm	72 rats (Mg–Y-Nd-HRE 36 rats) (Titanium 36 rats)	femur	sections parallel to the long axis of the implants	24 weeks	Differential blood count from blood sample obtained at sacrifice, IL-6 enzyme-linked immunosorbent assay, histology	systemic inflammatory reactions (lobulated neutrophil granulocytes, stab neutrophils, lymphocytes, eosinophil granulocytes or basophil granulocytes), IL-6 enzyme
**Erdmann et al.** [51]	2010	MgCa0.8 (Stainless steel 316L screws as control) Shape: screws Diameter: 4 ​mm Length: 6.0 ​mm Screw head: 8.0 ​mm	40 rabbits (MgCa0.8 24 rabbits) (Stainless steel 16 rabbits)	tibia	part of muscle adjacent to the screw head	8 weeks	histology, immunohistochemical staining	macrophages, giant cells, heterophil granulocytes, lymphocytes, B and T-lymphocytes
**Bondarenko et al.** [52]	2011	MgCa0.8 (Titanium, PLA group as control) Shape: N/A Diameter: 2.5 ​mm Length: 25 ​mm	9 rabbits (MgCa0.8 5 rabbits) (Titanium 2 rabbits) (PLA 2 rabbits)	tibia	popliteal lymph node	6 months	lymph node histology and immunohistochemistry	heterophiles, B-cells, T-cells, histiocytes
**Dziuba et al.** [53]	2012	ZEK100 (Sham group as a control) Shape: cylindrical Diameter: 2.5 ​mm Length: 25 ​mm	10 rabbits (7 animals-implant in both legs) (3 animals-one leg implant, other sham)	tibia	bone specimen containing implant	12 months	histology	macrophages, foreign body giant cells
**Willbold et al.** [54]	2013	RS66 Mg alloy Shape: cylindrical Diameter: 3 ​mm Height: 5 ​mm	30 rabbits	femur	bone sample	8 weeks	histology	macrophages, neutrophils
**Reifenrath et al.** [55]	2013	ZEK100 Mg alloy Shape: screws Head diameter: 8.0 ​mm Length: 5.0 ​mm	6 rabbits	tibia	muscle part directly adjacent to the screw head	6 weeks	Immunohistochemical staining	fibrous encapsulation, macrophages, giant cells and heterophil granulocytes, B- and T- lymphocytes
**Waizy et al.** [56]	2014	MgYREZr Shape: screws Shaft diameter: 2.0 ​mm Bore diameter: 1.3 ​mm Length: 20 ​mm	15 rabbits	femur	sections of bone perpendicular to the implant	12 months	histology	fibrous encapsulation
**Pichler et al.** [19]	2014	ZX50 WZ21 Shape: cylindrical pins Diameter: 1.6 ​mm Length: 8 ​mm	18 rats (6 rats sham group) (6 rats ZX50) (6 rats WZ21)	femur	ND	24 weeks	phagocytic assay, flow cytometry analysis	neutrophil granulocytes
**Willbold et al.** [57]	2015	Mg–Ce, Mg–La, Mg–Nd Shape: cylinders Diameter: 2.99 ​± ​0.01 ​mm Length: 5.00 ​± ​0.02 ​mm	9 rabbits (3 rabbits Mg–Ce) (3 rabbits Mg–La) (3 rabbits Mg–Nd)	femur	bone sample	4 weeks	histology	foreign body reaction, encapsulation
**Rossig et al.** [58]	2015	LAE442 magnesium-based alloy (stainless austenitic steel as a control) Shape: nails/screws, Diameter: 9 mm/3.5 ​mm Length: 130 mm/15–40 ​mm	10 sheep	tibia	bone sample	24 weeks	histology, blood sample	lymphocytes, macrophages, fibroblasts
**Tie et al.** [59]	2016	Mg–1Sr alloy, Pure Mg, (sham as a control) Shape: plates/screws	18 rabbits (6 ​Mg–1Sr alloy) (6 pure Mg) (6 sham)	femur	muscle perpendicular to the implantation site, spleen, kidney, liver	16 weeks	histopathology, haematology	T-cells, red blood cells, white blood cells, albumin, LDH, liver enzymes, bilirubin
**Diekmann et al.** [60]	2016	MgYREZr (Ti6Al4v as a control) Shape: screws Diameter: 2.6 ​mm Length: 10 ​mm Thread pitch: 0.8 ​mm	36 rabbits (18 rabbits MgYREZr) (18 rabbits Ti6Al4v control)	tibia	bone sample	24 weeks	histology	macrophages, granulocytes
**Angrisani et al.** [61]	2016	LAE442 magnesium-based alloy Shape: cylinders Diameter: 2.5 ​mm Length: 25 ​mm	8 rabbits	tibia	bone sample	9 months to 3.5 years	Histology, autopsy	giant cells, macrophages, eosinophilic infiltrates, eosinophilic granulocytes
**Wang et al.** [62]	2017	High purity Mg (Ti screws as a control) Shape: screws Diameter: 3 ​mm Length: 8 ​mm	64 rabbits	tibia and femur	bone sample	16 weeks	histology	macrophages, TGF beta 1
**Kim et al.** [63]	2018	Mg–Ca–Zn alloy (Polymeric mixture as a control) Shape: plates and screws Length, width, thickness: 24.5 ​mm ​× ​5.00 ​mm x 1.35 ​mm	6 male beagles	zygomatic bone	bone-implant interspace	4 weeks	Biochemistry, complete blood count, blood coagulation panels, histology	white blood count, macrophages, polymorphonuclear cells, lymphocytes, plasma cells, giant cells
**Rahmati et al.** [64]	2021	Mg–Ca–Zn (ZX00) alloy (Sham as a control) Shape: pins Diameter: 1.6 ​mm Length: 8 ​mm	12 rats	femur	bone sample	10 days	Enzyme histochemical analysis, immunohistochemistry	Osteoblast and osteoclast balance, M1 and M2 macrophages

ND; not done, N/A; not available.

Erdmann et al. evaluated the Mg–Ca0.8 alloy in tibia of 40 rabbits. Histology of muscle adjacent to the screw showed a moderate number of macrophages and giant cells two weeks after surgery, while the number of heterophils detected was lower when compared to other cell types. During the first weeks of implantation, the number of macrophages was decreasing, however, at the end of the observation period at week 8 the number of macrophages, heterophils and giant cells increased again. Immunohistochemical evaluation showed that B-lymphocytes in the MgCa0.8 group decreased from week 2 to week 4 and week 6 while there was an increase after week 8. T-lymphocytes were also increased in the later stage at week 8 [51].

Bondarenko et al. implanted different implant materials (Mg–Ca0.8, titanium, PLA) into the tibia of 9 rabbits with a follow up period of 6 months in order to compare morphological changes in efferent lymph nodes. The most remarkable results of the histology and immunohistochemistry were morphological changes reflected as sinus histiocytosis (excessive number of macrophages), rare occurrences of follicular hyperplasia, heterophilic infiltration and the appearance of histiocytic apoptosis. They concluded that the immunological reactions to MgCa0.8 increased during the study, but it was not significantly different than in the control groups [52].

Dziuba et al. investigated the long-term *in vivo* degradation behavior and biocompatibility of the Mg alloy ZEK100 in 10 rabbits. Animals were sacrificed 9 and 12 months after implantation. Bone slices were histologically analyzed and showed a significantly increased number of macrophages and foreign body giant cells in the intramedullary cavity. Additionally, fibrous tissue and cartilage were observable in specimens from the 9 months group as well as fibrous capsule formation in some specimens [53].

In 2013, Willbold et al. investigated the biocompatibility of rapidly solidified Mg alloy RS66 as a temporary biodegradable metal by implanting into the femur of 30 rabbits. Histological analysis revealed no significant acute immune response in bones. Moreover, macrophage-specific MAC 397 staining showed no increased appearance of these cells in bone [54].

In a study by Reifenrath et al., ZEK 100 ​Mg alloy was implanted into the tibia of six rabbits. Immunohistochemistry was performed on muscle exposed to the screw and showed significant macrophage and B- and T-lymphocyte infiltration. After 4 and 6 weeks, an increased presence of giant cells and macrophages that were aggregated into granuloma-like formation was reported. Furthermore, they observed mild to moderate heterophilic infiltration together with increased presence of apoptotic bodies. The presence of peri-implant fibrosis, necrosis and tissue cavities, as well as infiltration of giant cells, B-cells and heterophil granulocytes was increasing over 6 weeks, while macrophages and T-cells were decreasing, although they were also present after six weeks in muscle tissue adjacent to screw [55].

Waizy et al. evaluated implantation of MgYREZr screws into the femur of 15 rabbits. Histological analysis of bone specimens showed fibrous tissue in the region around the implant. However, there was no presence of fibrous capsule after 12 months noted and no systemic inflammatory reaction was observed in any animal [56].

Pichler et al. investigated the immunological response of the biodegradable Mg implants ZX50 and WZ21 after implantation into rat femoral bones. Their evaluation included phagocytic assay with flow cytometric analysis from the rat blood samples that were collected immediately before pin implantation and then every 4 weeks until the 24th week. Phagocytic ability of neutrophil granulocytes was significantly decreased in the no-implant group at week 0, 4 and 8 post-operatively. However, after 12 weeks there was a decreased phagocytic ability in the WZ21 group in comparison to the no-implant group, and at later time points there was no difference between the groups up to the 24th week. They concluded that biodegradable Mg implants have a beneficial effect on the immune system in a growing rat model [19].

Willbold et al. (2015) implanted Mg–Ce, Mg–La and Mg–Nd cylinders in both femoral bones of 9 rabbits. After a follow-up period of 4 weeks, the animals were euthanized and general histology on bone specimens was performed. The authors concluded that there was no encapsulation or signs of a foreign body reaction present. The implants did not produce any systemic or local cytotoxic effects, which was demonstrated by clinical observations and histology. However, all of the tested implants showed slow corrosion without stimulation of bone growth in the area around the implant after 4 weeks [57].

In a study by Rössig et al., magnesium-based LAE442 nails were inserted into the intramedullary space of the tibia of ten sheep and compared with stainless steel. In the bone specimen histology of both groups after 24 weeks, fibrous tissue was present in the bone marrow cavity where the nail was inserted. Fibrous capsules were present in both groups, however, in the steel group, it was more prominent. Also, in two cases of the LAE442 group, accumulation of inflammatory cells, such as lymphocytes and macrophages, together with fibroblasts, was observable, whereas inflammatory reaction was not observed in the steel group [58].

Tie et al. used Mg–1Sr alloy and pure Mg plates and screws that were implanted into the femurs of 18 rabbits and follow up was until week 16. Histopathology of peri-implant muscle, spleen, kidney and liver together with testing of hematological, inflammatory, cardiac and hepatic responses of samples demonstrated that the implantation of Mg–1Sr alloy and pure Mg did not trigger significant inflammation, did not cause inflammatory infiltrates, and did not induce adverse effects [59].

In the study by Diekmann et al., 36 rabbits were implanted with MgYREZr and titanium screws for a period of up to 24 weeks. Histology of bone specimens that were stained with Toluidine blue showed no evidence of inflammation, fibrosis or necrosis in both the Mg and Titanium group. Only focal infiltration of macrophages and granulocytes was present in the tendon tissue in one section of the 4-week Mg group [60].

Angrisani et al. implanted LAE442 Mg alloy cylinders into the tibiae of eight rabbits. After 3.5 years, histological analysis of tibia samples embedded into Technovit 9100 showed single macrophages with small groups of giant cells around the implant. Autopsy revealed mild to moderate eosinophilic infiltrates in liver and spleen [61].

In 2017, Wang et al. used high purity Mg screws for promoting tendon graft incorporation into the bone tunnel in 64 rabbits over 16 weeks. Immunohistochemical staining was performed on bone samples using RAM 11 monoclonal antibodies for macrophages and TGF-beta1, latter playing a key role in wound healing, angiogenesis and immunoregulation. Results showed an increased number of RAM 11-positive cells at week 3, representing macrophages involved in the wound healing process, however, after 6 weeks, the number of macrophages drastically decreased. The number of transforming growth beta (TGF beta1) positive cells around the bone tunnels was higher in the Mg group at week 3 and downregulated at week 6 [62].

In the manuscript published by Kim et al., the authors implanted Mg–Ca–Zn alloy plates and screws in zygomatic bones of six beagles, over 4 weeks. Toluidine blue staining of the bone-implant interface revealed no significant difference between the Mg–Ca–Zn and a control group (polymeric mixture) regarding fibrosis, fatty infiltrates or inflammatory cells such as macrophages, polymorphonuclear cells, lymphocytes, plasma cells, or giant cells. Therefore, they concluded that the magnesium alloy did not trigger a clinically significant inflammatory response, which was also supported by laboratory blood tests on inflammatory markers [63].

In 2021, Rahmati et al. investigated early body response to Mg-based ZX00 alloy by transcortical implantation of pins into rat femur with follow-up period of 2, 5 and 10 days. Immunohistochemistry with use of primary antibodies on bone samples revealed increased expression of macrophage type 2 biological markers after 10 days in Mg group. Furthermore, immunohistochemical analysis of bone samples indicated decreased activity of alkaline phosphatase and Runt-related transcription factor 2 (biological markers for osteoblast and osteoclast activity) in Mg group, which suggests decreased osteoblast activity. In the end authors concluded that ZX00 enhance the expression of macrophage polarization *in vivo* [64].

### 3.2 Risk of bias assessment for animal studies

Studies included in qualitative synthesis were assessed for the risk of bias by using SYRCLE tool in Table 2. Animal studies that stated use of randomization for sequence generation within the selection bias were attributed “Low risk” of bias [50,51,53,54,[56], [57], [58], [59], [60],^63^], while authors who did not provide any details on whether they used randomization for sequence generation were attributed “Unclear risk” [19,52,55,61,62,64]. All of the studies included in the qualitative synthesis provided baseline characteristic similarity such as sex, age or weight of animals and were entitled “Low risk” of bias [19,[50], [51], [52], [53], [54], [55], [56], [57], [58], [59], [60], [61],^63^], except the study by Wang et al. where baseline characteristics were not mentioned [62]. Information on whether the allocation to the different groups were adequately concealed during experiment were not stated in any of the evaluated studies which represents an “Unclear risk” of bias. Random housing and blinding domains within the performance bias were entitled “Unclear risk” of bias for all animal studies as there was no information provided on housing randomization and investigators/caregivers blinding approach. Only one study was entitled “High risk” for the domain of incomplete outcome within the attrition type of bias, due to reported death of two animals during the experiment [56]. Moreover, “Low risk” was attributed to all of the studies for the reporting bias as well as for the other sources of bias.

**Table 2:** Risk of bias assessment for animal studies (SYRCLE tool) [49].

Author	Selection bias			Performance bias		Detection bias		Attrition bias	Reporting Bias	Other
	Sequence generation	Baseline characteristics	Allocation concealment	Random housing	Blinding	Random outcome assessment	Blinding	Incomplete outcome	Selective outcome reporting	Other sources of bias
**Castellani et al.** [50]	Low risk	Low risk	Unclear risk	Unclear risk	Unclear risk	Low risk	Unclear risk	Low risk	Low risk	Low risk
**Erdmann et al.** [51]	Low risk	Low risk	Unclear risk	Unclear risk	Unclear risk	Low risk	Unclear risk	Low risk	Low risk	Low risk
**Bondarenko et al.** [52]	Unclear risk	Low risk	Unclear risk	Unclear risk	Unclear risk	Low risk	Unclear risk	Low risk	Low risk	Low risk
**Dziuba et al.** [53]	Low risk	Low risk	Unclear risk	Unclear risk	Unclear risk	Low risk	Unclear risk	Low risk	Low risk	Low risk
**Willbold et al.** [54]	Low risk	Low risk	Unclear risk	Unclear risk	Unclear risk	Low risk	Unclear risk	Low risk	Low risk	Low risk
**Reifenrath et al.** [55]	Unclear risk	Low risk	Unclear risk	Unclear risk	Unclear risk	Low risk	Unclear risk	Low risk	Low risk	Low risk
**Waizy et al.** [56]	Low risk	Low risk	Unclear risk	Unclear risk	Unclear risk	Low risk	Unclear risk	High risk	Low risk	Low risk
**Pichler et al.** [19]	Unclear risk	Low risk	Unclear risk	Unclear risk	Unclear risk	Low risk	Unclear risk	Low risk	Low risk	Low risk
**Willbold et al.** [57]	Low risk	Low risk	Unclear risk	Unclear risk	Unclear risk	Low risk	Unclear risk	Low risk	Low risk	Low risk
**Rossig et al.** [58]	Low risk	Low risk	Unclear risk	Unclear risk	Unclear risk	Low risk	Unclear risk	Low risk	Low risk	Low risk
**Tie et al.** [59]	Low risk	Low risk	Unclear risk	Unclear risk	Unclear risk	Low risk	Low risk	Low risk	Low risk	Low risk
**Diekmann et al.** [60]	Low risk	Low risk	Unclear risk	Unclear risk	Unclear risk	Low risk	Unclear risk	Low risk	Low risk	Low risk
**Angrisani et al.** [61]	Unclear risk	Low risk	Unclear risk	Unclear risk	Unclear risk	Low risk	Unclear risk	Low risk	Low risk	Low risk
**Wang et al.** [62]	Unclear risk	Unclear risk	Unclear risk	Unclear risk	Unclear risk	Low risk	Unclear risk	Low risk	Low risk	Low risk
**Kim et al.** [63]	Low risk	Low risk	Unclear risk	Unclear risk	Unclear risk	Low risk	Unclear risk	Low risk	Low risk	Low risk
**Rahmati et al.** [64]	Unclear risk	Low risk	Unclear risk	Unclear risk	Unclear risk	Low risk	Unclear risk	Low risk	Low risk	Low risk

## 4 Discussion

### 4.1 Do different types of Mg-based implants in terms of shape, size and alloying system cause different extent of immune response?

This systematic review supports previous findings which demonstrated that Mg-based implants are not biologically inert, and thus initiate an immune response when placed in living bone. Differences in the degree of implant degradation kinetics have shown to have an influence on the consistency of immunological response. This is reflected by the presence of immune cells observed by histological methods and can be supported by prior knowledge that chronic and granulation phases of an inflammatory response are dependent on the implant degradation rate [46]. Theoretically, systemic inflammatory reactions are possible, however, thorough investigation of the literature suggests that Mg-based implants do not initiate a systemic inflammatory response and currently, there is no *in vivo* study that actually reports this reaction. Mg-based implants are biocompatible, but with different degrees of inflammatory response, that certainly proved to be non-adverse [19,[50], [51], [52], [53], [54], [55], [56], [57], [58], [59], [60], [61], [62], [63], [64]].

#### 4.1.1 Implant physical properties in contrast to immune response

Several studies included in this review reported that no significant immunological event occurred in their experiments in terms of either increased presence of immune cells, foreign body reaction, fibrous capsule formation, or any other immunologically significant event [19,50,54,57,59]. However, these findings were contrary to the results reported by other studies [[51], [52], [53],^55^,^56^,^58^,[60], [61], [62], [63], [64]]. This indicates that different types of Mg-based implants in terms of shape, size and alloying system may cause variations in the intensity and time duration of an inflammatory response and wound healing process. Furthermore, differences can be explained by differences in the animal model, sex, age, implantation site, as well as different time points of performing laboratory and histology analysis. Another important aspect to be considered is the inflammatory response to the surgical procedure and localization. Based on the level of tissue damage, the intensity of inflammation may differ. Moreover, localization of implant insertion into different bone types and regions plays a fundamental role. Depending on the bone type (flat or long bones) and bone region (diaphysis or metaphysis) vascularization might support and improve healing process due to neutrophil's proximity to the damaged tissue area.

#### 4.1.2 Time point of immune cells activation

In the study by Erdmann et al., biocompatibility tests revealed that the chronic inflammatory response (macrophages, FBGCs formation) was minor in the first few weeks, but certainly increased around the 8th week. Also, humoral immunity reactions indicated by the presence of B-lymphocytes, was reported [51]. Another late increase of white blood cells was reported in the study by Dziuba et al. Nine months after Mg implantation, the number of macrophages and FBGCs was significantly increased in the intramedullary cavity. This study together with the study by Rossig et al. were the only studies that reported complete formation of fibrous capsule [53,58]. Late presence of inflammatory cells was also noted by Rossig et al. [58] after 24 weeks, as well as by Angrisani et al. after 3.5 years [61]. However, the studies by Reifenrath et al. and Wang et al. reported that after an initial increase of macrophages, there was a decrease in their presence after 6 weeks [55,62]. These findings support previous knowledge that the lifetime of macrophages on an implant surface can be from days to weeks and months [23]. These cells are considered to be the most important cells in chronic inflammation because of their secretion of biologically active products such as neural proteases, chemotactic factors, reactive oxygen metabolites, coagulation factors, growth promoting factors and cytokines [27]. However, it is not known if FBGCs remain active during their lifetime with lysosomal secretion, which can have an effect on implant biodegradation. These observations indicate that the residual implant material is not inert and triggers an increased presence of macrophages as well as FBGCs formation.

### 4.2 Are there missing links to properly understand immunological reactions upon implantation and degradation of Mg-based implants?

Importantly, the literature search highlights the absence of severe inflammatory reactions upon Mg implantation. Sporadic and moderate immune response that were noticed in some of the reviewed studies indicate the need for further research regarding the kinetics of immune response upon Mg implant degradation.

#### 4.2.1 Macrophages’ role in immune responses

One of the most important cells in the process of immune responses appeared to be macrophages. Their interaction with biomaterials needs to be elucidated for a better comprehension of its effect on degradation, especially their exact role in biodegradation of implants, since it has been previously demonstrated that esterase secreted by macrophages can mediate polycarbonate-urethane biodegradation [65]. Another aspect is the characterization of the exact role of M1 vs M2 macrophages in mediating inflammatory response.

#### 4.2.2 Immune response of biodegradable, in comparison to permanent implants

Both permanent and biodegradable metal implants release ions after implantation and activate the immune system by forming protein complexes, which can later induce hypersensitivity [27]. These conglomerates of ions, especially in alloys with rare earth elements, can be found even in regions which are far from the bone-implant interface [66]. Qiao et al. demonstrated an immunomodulatory role of Mg^2+^ in the early bone healing phase [67]. Macrophages are stimulated by transient receptor potential cation channel member 7 (TRPM7), in order to generate a specific pro-osteogenic immune microenvironment [67,68]. Moreover, Mg^2+^ may affect the osteogenic differentiation of osteoblast lineage by activating different cells within the bone and stimulate early osteoclast differentiation [67]. In terms of hydrogen gas formation recent findings indicate that H_2_ release after Mg degradation can decrease expression of several pro-inflammatory factors such as TNF-α, IL-6, IL-1β, CCL2, IL-10, TNF-γ, IL-12, CAM-1, HMGB-1, PGE2, and nuclear factor-κB (NF-κB) [69]. Furthermore, Roth et al. showed that magnesium implant debris particles do not induce exaggerated immune reaction or any immunosuppressive properties [70]. Therefore, the long-term effect of ions released by biodegradable implants should be deeply elucidated. Moreover, a comparison of ion release between biodegradable and permanent implants is urgently needed, especially focusing on permanent implants and their effect on triggering hypersensitivity reactions [40]. However, based on current literature, Mg-based and permanent implants show similar immunological properties in terms of adverse immunological response absence. Nevertheless, Mg-based implants possess promising properties in terms of biodegradation, avoidance of stress shielding, and osteogenic differentiation which can be caused by permanent implants, in addition to the absence of late-stage infection due to their degradation times. Therefore, the understanding of biodegradation's long-term impact on the immunological system will facilitate in the patient's choice to undergo treatment with a new medical device.

#### 4.2.3 Limitations

The aim of this systematic review was to gather all of the information from the available *in vivo* studies with Mg-based implants regarding their induction of inflammatory reaction. There are several limitations of the reviewed studies that need to be highlighted. First, the study duration between the proposed *in vivo* experiments varied and only one study was longer than 12 months and demonstrated the late presence of inflammatory cells even after 3.5 years. Another limitation is that only 16 studies provided more information on biocompatibility and host immunological reactions of biodegradable Mg implants in the searched time frame, and there was no clinical study done in humans that tested this topic in more detail. Moreover, implant material composition differed between the studies, however, Mg was the most abundant element in all of them. Besides that, different animal species, ages, sexes and implantation sites were used, which constitutes another important limitation.

Even though baseline characteristics were entitled low risk within the SYRCLE risk of bias assessment tool, the actual difference of characteristics between the studies may be the culprit for different degradation kinetics, which in the end effects the extent of immune response. Moreover, lack of reporting on domains within the performance bias as well as possible study blinding protocols assessed by SYRCLE tool represent another important limitation within the evaluated studies. Therefore, more detailed explanations of animal experiments section within the preclinical studies are needed in future for better comparison of preclinical research outcomes. This is especially emphasized when different animal models are used, due to possible differences in mechanisms of immune response to degrading Mg-implants. Moreover, to the best of our knowledge there is no study that compare the difference in immune system response between the animal species, with a connection to implant testing. Nevertheless, this systematic review considered all of the limitations and serves as a general review of Mg-based implant's *in vivo* host reaction.

#### 4.2.4 Methodological approach for future studies

Histology together with immunohistochemistry as a conventional method proved to be useful for evaluating immunological reaction. However, other methods should be also considered in order to obtain a wider set of information on immunogenicity of previously discussed implants. Previously reviewed studies highlighted the importance of evaluating M1 and M2 macrophage polarization, function and presence, as their absence on implant surface and in surrounding bone tissue indicates the resolution of foreign body reaction. Both M1 and M2 phenotypes showed to have beneficial roles in osteogenesis [67]. Moreover, macrophages have been considered as a major cell in bone healing and immune response to biomaterials due to their numerous roles in bone homeostasis [71]. Li et al. reported that the release of Mg^2+^ from Mg–Si–Ca alloy activates the macrophage lineage, induces the recruitment of mesenchymal stem cells and stimulates osteogenic differentiation [68]. Non-invasive optical methods showed to be a useful technique for macrophage imaging in animal models, in particular bioluminescence and intravital microscopy [72]. Moreover, the use of these techniques may avoid animal euthanasia at early time points and provide a usefulness in long-term research. The combination of optical methods with histology, immunohistochemistry, enzyme histochemistry and gene expression analysis in a long-term study would help to elucidate processes during initiation, course and resolution of the inflammatory response to Mg-based implants. Consequently, it would contribute to more comparability of *in vivo* studies as well as in determining the exact timeline of inflammatory response to tested implants by detailed evaluation of parameters previewed in Fig. 1.

## 5 Conclusion

The findings from studies included in this review have certain variability, however, the most important finding is that Mg implants did not cause severe inflammatory reactions in any of the included studies, and their mild to moderate inflammatory potential can be confirmed by this systematic review. Besides that, it is obvious that there were discrepancies between the studies regarding the timepoints of actual inflammatory reaction, which may be attributed to different degradation kinetics of tested implants. The exact mechanism of the implant biodegradation effect on the immune system should be deeply investigated and therefore, detailed, long-term studies with the use of more complex techniques on the immunological response upon Mg implant degradation are urgently needed. Finally, better understanding will facilitate the decision of patients to undergo new device implantation.

## Declaration of competing interest

The authors declare that they have no known competing financial interests or personal relationships that could have appeared to influence the work reported in this paper.
